# Effect of COVID-19 changes on outcomes and socioeconomic disparities following metabolic and bariatric surgery

**DOI:** 10.1007/s00464-024-11212-z

**Published:** 2024-09-12

**Authors:** Shushmita M. Ahmed, Alexandra Johns, Leah Timbang, Annie Wang, Navneet Kaur Singh, Victoria Lyo, Mohamed Ali

**Affiliations:** 1 Department of Surgery, University of California, Davis, 2335 Stockton Blvd, 6th Floor, Sacramento, CA 95817 USA; 2 Center for Metabolic and Alimentary Science, University of California, Davis, Sacramento, USA; 3 School of Medicine, University of California, Davis, Sacramento, USA

**Keywords:** COVID-19, Socioeconomic status, Bariatric surgery, Outcomes, Follow-up

## Abstract

**Background:**

We previously showed worse outcomes among lower socioeconomic status (SES) groups following metabolic/bariatric surgery (MBS). In light of healthcare changes in response to COVID-19, this study aims to evaluate post-pandemic MBS outcomes and determine if prior socioeconomic disparities persisted in the post-COVID era.

**Methods:**

A retrospective chart review of patients undergoing primary Roux-en-Y gastric bypass (RYGB) and sleeve gastrectomy (SG) between 2015 and 2022 was performed. Patients were stratified into pre- and post-COVID groups. Post-COVID cohort was further stratified into high (HT) and low (LT) tier status based on Distressed Communities Index, a geocoded composite measure of SES. Preoperative characteristics and postoperative outcomes were compared between pre- and post-COVID cohorts, as well as between post-COVID HT and LT groups.

**Results:**

Of 709 patients, 82.9% were pre-COVID and 17.1% were post-COVID. Post-COVID cohort had greater rate of public insurance (46% vs. 37%, *p* < 0.001), longer wait time to surgery (mean 358 ± 609.8 days vs 241.9 ± 368.5 days, *p* = 0.045), and were more likely to undergo RYGB (69% vs. 56%, *p* = 0.010). Post-COVID patients also had lower risk of any complications on multivariable analysis (OR 0.599, 95% CI 0.372–0.963), had higher follow-up rates at post-discharge (95.8% vs 79.7%, *p* < 0.005), 6-month (93% vs. 82%, *p* < 0.001) and 12-month visits (75% vs. 63%, *p* = 0.005), and lost more weight at 12 months (67% excess weight loss (%EWL) vs. 58%EWL, *p* = 0.002). Among post-COVID HT and LT cohorts, previously seen disparities in complications were no longer seen. Finally, there were no differences in weight or follow-up rates between post-COVID HT and LT.

**Conclusions:**

Post-COVID changes to MBS care have resulted in improved short-term outcomes and reduced disparities for patients of lower SES. Further studies are needed to identify these positive factors to perpetuate practice patterns that optimize care for patients of all socioeconomic status.

The coronavirus disease of 2019 (COVID-19) led to drastic changes in healthcare access and delivery. The infectious disease burden necessitated the reallocation of resources including staff, equipment, and even physical spaces to accommodate the influx of patients [1]. As little was known about the transmission of the virus in the early stages of the pandemic, elective surgeries were often suspended or regulated according to medical necessity [1, 2]. This led to a notable decrease in operations, with a nadir of 27% fewer procedures performed from 2019 to 2020 [3]. Similarly, performance of metabolic/bariatric surgeries (MBS) dropped by 12.1% [4–6]. What is more, post-pandemic patient and procedure type shifted, favoring more sleeve gastrectomy and younger patients [4, 5].

One major factor affecting access to and outcomes following MBS is socioeconomic status (SES) [7–10]. SES can be difficult to calculate as it is multifactorial and requires information beyond the medical record. Distressed Communities Index (DCI) is a geocoded composite score that provides a more comprehensive measure of SES [11]. DCI measures community economic well-being by accounting for the following: community education rates, poverty rates, income, housing status, employment status, rate of change in employment, and business growth. Several studies have used DCI for surgical risk stratification and to predict surgical outcomes [12–14]. Our group previously used DCI to evaluate differences in outcomes between high and low resource communities following MBS, revealing worse 30-day complication rates and increased prevalence of weight recurrence among patients from distressed communities compared to their more prosperous counterparts [15, 16].

Few studies have specifically reported on the outcomes in patients undergoing MBS in the post-COVID era, and none have evaluated the effect of SES. Thus, given the change in the healthcare landscape and the known discrepancies seen among our patients, we aimed to evaluate post-pandemic MBS outcomes and determine if these disparities between high and low resourced communities persisted in the post-pandemic era.

## Methods

A retrospective cohort analysis of all patients ≥ 18 years undergoing primary laparoscopic Roux-en-Y gastric bypass (RYGB) or sleeve gastrectomy (SG) between 2015 and 2022 was performed at a single academic institution following approval by the intuitional review board. Clinical data were extracted from the electronic medical record (EMR): demographics, preoperative characteristics, wait time to surgery from initial consultation, 30-day complication rates, weight, and postoperative follow-up rates. Telehealth visits utilized self-reported weights. Patients undergoing surgery before March 1, 2020 were placed into the pre-pandemic cohort, while those with surgery dates after March 1, 2020 were placed into the post-pandemic cohort.

### Distressed communities index

DCI defines community borders by zip code and assigns each community a score from 0 (no distress) to 100 (severe distress). Communities are then ranked into 5 categories: distressed, at risk, mid-tier, comfortable, and prosperous [11]. Zip codes were obtained from the EMR to score and categorize each patient into their respective DCI category. Patients were then stratified into high tier DCI status (HT), which includes mid-tier, comfortable and prosperous communities, and low tier DCI status (LT) which includes at-risk and distressed communities.

### Weight calculations

Preoperative weight was reported as the body mass index (BMI) on the date of surgery. Postoperative weight loss was reported in standardized measure of percent excess weight loss (%EWL) and percent total weight loss (%TWL) [17]. Excess weight was calculated as the difference between initial weight and ideal body weight, with ideal body weight calculated based on a BMI of 25 kg/m^2^. Weight loss was recorded at 6- and 12-month postoperative time points.

### Follow-up visits

In addition to the standard 6-month and 12-month postoperative follow-up rates, we reported short-term (30-day) follow-up rates for all of our patients. All patients at our institution are scheduled for a post-discharge telephone visit 2–3 days following discharge and an in-person 2-week postoperative visit. Six- and 12-month visits were in person or via telemedicine based on patient availability and preference.

### Statistics

Statistical analysis was done using R Studio Software, version 4.1.2 (R Studio Team, 2020, PBC, Boston, MA). Student’s t test was used to compare continuous variables and Chi-squared analyses were used to compare categorical variables between HT and LT groups. Multivariable linear regressions were performed for continuous dependent variables, while multivariable binary regressions were performed for nominal dependent variables. Both types of analyses controlled for age, sex, race, type of surgery, preoperative BMI, American Society of Anesthesiologists (ASA) status, and type of insurance.

## Results

Of 709 patients undergoing primary MBS, 588 (82.9%) underwent surgery prior to March 1, 2020, and 121(17.1%) underwent surgery after the start of the COVID-19 pandemic. Demographic data are summarized in Table 1. Overall, mean age was 48.6 ± 12.2 years and 76.0% were women. There were no differences between pre- and post-COVID groups in age, sex, race, or ethnicity. Post-COVID patients were more likely to have public insurance (46.3% vs 36.6% pre-COVID patients, *p* < 0.001), with higher proportions of Medicaid and military health insurance. DCI breakdown and percentage of HT vs LT patients were similar between pre- and post-COVID groups.

**Table 1:** Demographic data of all patients undergoing primary bariatric surgery, stratified by pre- and post-COVID status

	Overall *n* = 709	Pre-COVID *n* = 588	Post- COVID *n* = 121	*p*-value
Participant characteristics				
Age in years, mean (SD)	48.6 (± 12.2)	48.7 (± 12.3)	48.3 (± 12.7)	0.715
Min. Age	18	18	19	
Max. Age	80	80	78	
Female, (%)	76.0%	76.4%	74.4%	0.627
DCI Category, *n* (%)				
5	197 (28.1%)	165 (28.1%)	32 (26.4%)	
4	187 (26.4%)	151 (25.7%)	36 (29.8%)	
3	135 (19.0%)	115 (19.6%)	20 (16.5%)	
2	149 (21.0%)	125 (21.3%)	24 (19.8%)	
1	33 (4.7%)	27 (9.4%)	6 (5.1%)	
NA	8 (1.1%)	5 (0.9%)	3 (2.5%)	
DCI Group, *n* (%)				0.300
High Tier (DCI Category 3–5)	519 (73.2%)	431 (73.3%)	88 (72.7%)	
Low Tier (DCI Category 1–2)	182 (25.7%)	152 (25.9%)	30 (24.8%)	
Race Breakdown, *n* (%)				0.300
White	497 (70.1%)	414 (70.4%)	83 (68.6%)	0.517
African American	81 (11.4%)	66 (11.2%)	15 (12.4%)	
Hispanic	80 (11.3%)	65 (11.1%)	15 (12.4%)	
Native Hawaiian or Pacific Islander	9 (1.3%)	8 (1.4%)	1 (0.8%)	
Asian	12 (1.7%)	8 (1.4%)	4 (3.3%)	
American Indian or Alaska Native	9 (1.3%)	7 (1.2%)	2 (1.7%)	
Other or Undetermined	21 (2.7%)	20 (3.4%)	1 (0.8%)	
Hispanic Ethnicity, (%)	17.5%	17.3%	17.4%	0.302
Insurance Breakdown, *n* (%)				** < 0.001**
All Private	438 (61.8%)	373 (63.4%)	65 (53.8%)	
All Public	271 (38.2%)	215 (36.6%)	56 (46.3%)	

Bold value indicates statistical significance (*p* < 0.05)

Statistics comparing pre-COVID to post-COVID cohorts

*SD* Standard deviation

*DCI* Distressed Communities Index

Table 2 shows the demographic data of the post-COVID groups. Mean age was 48.2 ± 12.7 years and 74.4% of patients were female. When stratified by DCI tier, 72.7% (88) were HT and 24.8% (30) were LT. Three patients had unknown DCI status. HT and LT groups were similar in age, sex, race, and ethnicity. Post-COVID LT patients had a higher rate of public insurance than post-COVID HT (66.7% LT vs 36.4% HT, *p* < 0.001) counterparts.

**Table 2:** Demographic data of post-COVID patients, stratified by high and low tier DCI groups

	Overall *n* = 121	High Tier *n* = 88	Low Tier *n* = 30	*p*-value
Participant characteristics				
Age in years, mean (SD)	48.2 (± 12.7)	49 (± 12.6)	44.4 (± 12.5)	0.085
Min. Age	19	19	26	
Max. Age	78	78	65	
Female, (%)	74.4%	73.9%	82.7%	0.527
DCI Category, *n* (%)				
5	32 (26.5%)	32 (36.3%)	-	
4	36 (29.8%)	36 (40.9%)	-	
3	20 (16.53%)	20 (22.7%)	-	
2	24 (19.8%)	-	24 (80.0%)	
1	6 (5%)	-	6 (6.8%)	
NA	3 (2.5%)	-	-	
Race Breakdown, *n* (%)				0.156
White	83 (68.6%)	59 (67.1%)	21 (63.6%)	
African American	15 (12.4%)	14 (14.9%)	1 (3.0%)	
Hispanic	15 (12.4%)	9 (10.2%)	6 (18.2%)	
Native Hawaiian or Pacific Islander	1 (0.8%)	1 (1.1%)	0 (0.0%)	
Asian	4 (3.31%)	4 (4.6%)	0 (0.0%)	
American Indian or Alaska Native	2 (1.7%)	1 (1.1%)	1 (3.0%)	
Other or Undetermined	1 (0.8%)	-	1 (3.0%)	
Hispanic Ethnicity, (%)	21 (17.4%)	17.1%	18.2%	0.135
Insurance Breakdown, *n* (%)				** < 0.001**
All Private	64 (52.9%)	56 (63.6%)	8 (24.2%)	
All Public	54 (44.63%)	32 (36.4%)	22 (66.7%)	

Bold value indicates statistical significance (*p* < 0.05)

Statistics comparing high tier DCI to low tier DCI groups

*SD* Standard deviation

*DCI* Distressed Communities Index

Post-COVID patients had significantly longer wait time from initial consultation to surgery date than pre-COVID patients (358 ± 610 days post-COVID vs 242 ± 369 days pre-COVID, *p* = 0.045). This was true even when controlling for age, gender, race, ethnicity, insurance type, BMI, ASA, and type of surgery (pre- vs post-COVID coefficient 110.053, *p* = 0.012). Post-COVID patients also had slightly lower preop BMI at time of surgery (42.8 ± 6.2 kg/m^2^ post-COVID vs. 44.3 ± 7.4 kg/m^2^ pre-COVID, *p* < 0.023), which while statistically significant was not clinically significant. Both groups had similar preoperative weight loss and ASA status. Finally, the post-COVID group had higher rates of RYGB than pre-COVID cohort (69.4% RYGB post-COVID vs. 56.3% pre-COVID, *p* = 0.010) (Table 3).

**Table 3:** Preoperative characteristics and type of surgery, stratified by pre- and post-COVID status

	Overall *n* = 709	Pre-COVID *n* = 588	Post-COVID *n* = 121	*p*-value
Preoperative characteristics				
Wait time to surgery,^a^ days	261.7	241.9	358	**0.045**
Mean (SD)	(± 421.3)	(± 368.5)	(± 609.8)	
Min. Days	19	19	59	
Max. Days	4315	3370	4315	
Excess Weight, kg	53.6 (± 21.2)	54.2 (± 21.6)	50.4 (± 18.9)	0.053
Mean (SD)				
Preop Weight Loss,^b^ %TBW	3.2% (± 13.0%)	3.3% (± 14.1)	2.89% (± 5.2%)	0.747
Mean (SD)				
Preoperative BMI,^c^ kg/m^2^	44.0 (± 7.3)	44.3 (± 7.4)	42.8 (± 6.2)	**0.023**
Mean (SD)				
Min. BMI	32.1	32.1	32.1	
Max. BMI	79.9	79.9	69.5	
ASA Status *n* (%)				0.632
ASA—2	85 (12.0%)	73 (12.4%)	12 (9.9%)	
ASA—3	616 (86.9%)	509 (86.6%)	107 (88.4%)	
ASA—4	8 (1.1%)	6 (1.0%)	2 (1.7%)	
Type of Surgery *n* (%)				**0.010**
Sleeve Gastrectomy	294 (41.5%)	257 (43.7%)	37 (30.6%)	
Roux-en-Y Bypass	415 (58.5%)	331 (56.3%)	84 (69.4%)	

Bold values indicate statistical significance (*p* < 0.05)

Statistics comparing pre-COVID to post-COVID cohorts

*SD* Standard deviation

*%TWL* Percent total body weight loss

^a^From initial consultation visit

^b^From initial visit to date of surgery

^c^BMI at time of surgery

Among post-COVID patients, HT and LT patients had similar preoperative characteristics including preoperative BMI, wait time to surgery from initial consultation, and ASA profiles (Table 4). LT patients trended toward greater rates of RYGB (83.3% LT vs. 64.8% HT), although this was not statistically significant with *p* = 0.094.

**Table 4:** Preoperative characteristics and surgery type of post-COVID patients, stratified by high and low tier DCI groups

	Overall *n* = 121	High Tier *n* = 88	Low Tier *n* = 30	*p*-value
Preoperative characteristics				
Wait time to surgery,^a^ days	359.5	365.3	342.3	0.863
Mean (SD)	(± 617.3)	(± 616.3)	(± 630.3)	
Min. Days	59	59	5	
Max. Days	4315	4315	3608	
Excess Weight, kg	125.6 (± 26.6)	125.3 (± 27.4)	126.3 (± 24.5)	0.852
Mean (SD)				
Preop Weight Loss,^b^ %TBW	50.4 (± 19)	50.3 (± 20.2)	50.7 (± 15.5)	0.909
Mean (SD)				
Preoperative BMI,^c^ kg/m^2^	42.8 (± 6.32)	42.7 (± 6.59)	43 (± 5.55)	0.807
Mean (SD)				
Min. BMI	32.1	32.1	34.9	
Max. BMI	69.5	69.5	57.3	
ASA Status *n* (%)				0.525
ASA—2	12 (10.2%)	10 (11.4%)	2 (6.7%)	
ASA—3	104 (88.1%)	76 (86.4%)	28 (93.3%)	
ASA—4	2 (1.7%)	2 (2.3%)	0 (0.0%)	
Type of Surgery *n* (%)				0.094
Sleeve Gastrectomy	36 (30.5%)	31 (35.2%)	5 (16.7%)	
Roux-en-Y Bypass	82 (69.5%)	57 (64.8%)	25 (83.3%)	

Statistics comparing high tier DCI to low tier DCI groups

*SD* Standard deviation

*%TWL* Percent total body weight loss

^a^From initial consultation visit

^b^From initial visit to date of surgery

^c^BMI at time of surgery

Post-COVID patients trended toward shorter hospital length of stay (LOS) (1.7 ± 1.0 days post-COVID vs 1.9 ± 1.4 days pre-COVID, *p* = 0.076) but had similar rates of patients discharged by postoperative day 1 (POD1) (48.8% post-COVID vs 42.7% pre-COVID, *p* = 0.260). While most 30-day complication rates were similar between groups, univariate analysis showed significantly lower wound complication rates in the post-COVID cohort (0.8% post-COVID vs 5.4% pre-COVID, *p* < 0.001) (Table 5). However, when controlling for preoperative characteristics, this difference was no longer seen. Instead, multivariable binary logistic regression showed that post-COVID patients were half as likely to experience any complication, with an odds ratio of 0.599 [95% CI 0.372–0.963] (Table 6).

**Table 5:** Postoperative outcomes of pre- vs post-COVID cohorts

	Overall *n* = 709	Pre-COVID *n* = 588	Post-COVID *n* = 121	*p*-value
Discharge status				
Length of Stay, days	1.9 (± 1.3)	1.9 (± 1.4)	1.7 (± 1)	0.076
Mean (SD)				
Discharged on POD1, (%)	43.7%	42.7%	48.8%	0.260
30-Day Complications *n* (%)				
Any Complication	211 (29.8%)	182 (31.0%)	29 (24.0%)	0.109
Readmission	32 (4.5%)	27 (4.6%)	5 (4.1%)	0.820
Wound Complications	33 (4.7%)	22 (5.4%)	1 (0.8%)	** < 0.001**
Anastomotic Leak	0	0	0	−
Dehydration	131 (18.5%)	109 (18.5%)	22 (18.2%)	0.927
Death	2 (0.3%)	2 (0.3%)	0	0.158
Follow-up Attendance Rates, *n* (%)				
Post-discharge Telephone Visit	584 (82.4%)	470 (79.7%)	114 (95.8%)	** < 0.001**
2-Week Postop Visit	584 (82.4%)	586 (99.3%)	118 (99.2%)	0.308
6-Month Postop Visit	592 (83.5%)	480 (81.6%)	112 (92.6%)	** < 0.001**
12-Month Postop Visit	460 (64.9%)	369 (62.8%)	91 (75.2%)	**0.005**
6-Month Weight Loss				
% EWL, mean (SD)	49.3 (± 17.2)	48.3 (± 17.0)	53.6 (± 17.0)	**0.004**
% TWL, mean (SD)	19.9 (± /− 5.93)	19.6 (± /− 5.89)	20.9 (± 6.02)	**0.033**
12-Month Weight Loss				
% EWL, mean (SD)	59.8 (± /− 23.1)	58.1 (± /− 23.0)	66.7 (± /− 22.5)	**0.002**
% TWL, mean (SD)	24.4 (± /− 9.07)	23.9 (± /− 9.09)	26.4 (± /− 8.76)	**0.019**

Bold values indicate statistical significance (*p* < 0.05)

Statistics comparing pre-COVID to post-COVID cohorts

*SD* Standard deviation; *POD* = Postoperative day

*%EWL* Percent excess weight loss

*%TWL* Percent of total body weight loss

**Table 6:** Multivariable binary regression comparing postoperative complications of pre vs post-COVID patients. *Odds ratios represent post-COVID group*

	Odds Ratio	95% CI
Any Complication*	0.599	0.372–0.963
Readmission	0.471	0.156–1.419
Wound Complications	0.139	0.019–1.035
Dehydration	0.840	0.493–1.431

Control variables: Age, Gender, Race, Ethnicity, Insurance Type, BMI at Surgery, ASA, and Type of Surgery

*Odds ratios do not cross 1

Post-COVID patients also had statistically higher rates of follow-up, particularly at the post-discharge telephone (95.8% vs 79.7%, *p* < 0.001), 6-month postop (92.6% vs 81.6%, *p* < 0.001), and 12-month postop (75.2% vs 62.8%, *p* < 0.005) visits (Table 5). There was no difference in the 2-week follow-up visit rates, with both groups having over 99% attendance. Post-COVID patients also had greater weight loss at both 6-month (53.6 ± 17.0%EWL vs. 48.3 ± 17.0%EWL, *p* = 0.004) and 12-month (66.7 ± 22.5%EWL vs. 58.1 ± 23.0%EWL, *p* = 0.002) postop. On multivariable linear regression, again controlling for factors including preoperative BMI, and type of surgery, differences in %EWL and %TWL were only statistically significant at the 12-month time point (Table 7).

**Table 7:** Multivariable linear regression comparing postoperative weight loss of post-COVID (compared to pre-COVID) patients

	Coefficient	Std Error	*p*-value
6-Month Weight Loss			
%EWL at 6 months	0.0226	0.014	0.110
%TWL at 6 months	1.021	0.554	0.066
12-Month Weight Loss			
%EWL at 12 months	0.0497	0.023	**0.031**
%TWL at 12 months	2.195	0.944	**0.020**

Bold values indicate statistical significance (*p* < 0.05)

Control Variables Age, Gender, Race, Ethnicity, Insurance Type, BMI at Surgery, ASA, and Type of Surgery

*%EWL* Percent excess weight loss

*%TWL* Percent of total body weight loss

Table 8 compares postoperative outcomes among post-COVID patients stratified by HT and LT DCI groups. There were no differences in LOS (1.8 ± 0.95 days), and while there was a greater percentage of LT patients discharged on POD 1 (45.5% HT vs 63.3% LT), this was not statistically significant (*p* = 0.139). There were no differences in 30-day complication rates and follow-up rates were similar across all times points. Finally, there were no differences in weight loss between groups at 6 months (54.5 ± 17.9%EWL HT vs 50.6 ± 15.2%EWL LT, *p* = 0.313) and 12 months (65.6 ± 23.0%EWL HT vs 69.5 ± 21.4%EWL LT, *p* = 0.473) postoperatively.

**Table 8:** Postoperative outcomes high vs low tier post-COVID patients

	Overall *n* = 121	High Tier *n* = 88	Low Tier *n* = 30	*Odds Ratio*	*p*-value
Discharge Status					
Length of Stay, days	1.8 (± 0.95)	1.8 (± 0.94)	1.6 (± 1)	-	0.476
Mean (SD)					
Discharged on POD1, (%)	59 (48.8%)	40 (44.0%)	19 (63.3%)	-	0.139
30-Day Complications *n* (%)					
Any Complication	29 (24.0%)	23 (25.3%)	6 (20.0%)	0.648	0.497
Readmission	5 (4.10%)	4 (4.40%)	1 (3.30%)	0	0.996
Wound Complications	1 (0.800%)	1 (1.10%)	0 (0.00%)	*	> 0.999
Anastomotic Leak	0	0	0	*	*
Dehydration	22 (18.2%)	17 (18.7%)	5 (16.7%)	0.483	0.807
Death	0	0	0	*	*
Follow-up Attendance Rates, *n* (%)					
Post-discharge Telephone Visit	116 (95.9%)	84 (95.5%)	29 (96.7%)		0.809
2-Week Postop Visit	121 (100%)	88 (100%)	30 (100%)		0.103
6-Month Postop Visit	109 (92.4%)	83 (94.3%)	26 (86.7%)		0.334
12-Month Postop Visit	75.4%	75.0%	76.7%		0.199
6-Month Weight Loss					
% EWL, mean (SD)	53.3 (± 0.17)	54.5 (± 17.9)	50.6 (± 15.2)		0.313
% TWL, mean (SD)	20.9 (± 6.02)	21.0 (± 5.81)	20.9 (± 6.78)		0.966
12-Month Weight Loss					
% EWL, mean (SD)	66.6 (± 22.5)	65.6 (± 23.0)	69.5 (± 21.4)		0.473
% TWL, mean (SD)	26.4 (± 8.76)	25.6 (± 8.80)	28.7 (± 8.43)		0.154

Statistics comparing high tier DCI to low tier DCI groups

*SD* Standard deviation, *POD* Postoperative day

*%EWL* Percent excess weight loss

*%TWL* Percent of total body weight loss

*p < 0.05

## Discussion

COVID-19 has left its mark on healthcare systems world-wide. In addition to changes in MBS access and delivery, postoperative care protocols and telehealth have transformed the field [2, 18]. The burden of obesity makes bariatric patients particularly vulnerable—SES exacerbates this vulnerability [10, 19, 20]. Thus, in the face of major healthcare changes, our study characterizes outcomes in the post-COVID era with attention to disparities between SES groups.

One of the most notable adverse impacts of the pandemic is the reduction in the number of MBS procedures performed. This has led to delays in the care for patients with obesity [6, 21, 22]. Our study population is no exception; post-COVID status was an independent predictor for increased wait time to surgery, with average wait time 4 months longer than pre-COVID counterparts. Again, this is likely a reflection of prioritization of urgent procedures and limits of hospital capacity. Even with this limitation, our study is unique as we saw no differences in age or ASA status between groups [4, 5]. This suggests that despite delays, obesity management of sicker and older patients was not compromised.

This study did identify two notable preoperative differences between pre- and post-COVID groups. First, there was an increase in the prevalence of public insurance, rising from 36.6% pre-COVID to 46.3% post-COVID. This may be attributable to the expansion of Medicaid and laxity of eligibility criteria in response to the COVID-19 [23, 24]. Second, we saw an increase in prevalence of RYGB performed, from 56 to 69%. This is consistent with trends seen in the MBSAQIP registry; while overall prevalence of SG increased from 2019 to 2020, subgroup analyses found a 11% increase in the rate of RYGB among adults ≥ 19 years [4, 5]. This suggests that the post-COVID environment allowed for greater patient catchment without compromising operative technique.

Consistent with prior studies, there was no difference in LOS and complication rates were mostly similar between pre- and post-COVID groups [4, 6, 25]. The one exception was rate of any complication; multivariable analyses showed that post-COVID patients were half as likely to have any complication compared to the pre-COVID cohort, suggesting a protective effect of hospital protocols and practice patterns since the pandemic began. This may be due in part to increased accessibility to the health care team through telehealth, though the effect new policies aimed at infection prevention and earlier discharge are unknown. Further studies are required to determine additional factors allowing for the improvement of complication metrics.

Perhaps one of the most positive and pervasive changes incurred by the pandemic is the standardization of telehealth. While pre-pandemic studies demonstrated the safety and efficacy of telehealth for postoperative care, the demands of the pandemic catalyzed its widespread adoption [26, 27]. The impact of this is most apparent in our follow-up rates. Prior to the pandemic, 2-week postop visit had over 99% attendance rate, primarily due to required in-person wound evaluation. Visits with telehealth options, however, had less than 80% attendance. For example, although a post-discharge telephone visit has been a part of our practice since 2014, attendance prior to the pandemic was only 80%. Post-COVID attendance improved to 96%. This is likely due to new protocols enforcing remote visits (to satisfy isolation requirements), improvements in telehealth platform usability, and new billing codes facilitating visit compensation. Similar improvements in follow-up were seen at 6 and 12 months, likely due to telehealth accessibility and insurance coverage. Gould et al*.* showed insurance coverage to be a major barrier of long-term follow-up [28]. As aforementioned, Medicaid coverage was expanded and was less likely to be revoked, thus increasing the pool of insured patients. Given the rates of public insurance in our post-COVID cohort, future studies are needed to see whether these high rates of follow-up persist as Medicaid returns to its baseline coverage patterns.

Interestingly, while other studies predominantly report similar pre- and post-COVID weight loss following MBS, we saw greater 12-month weight loss in the post-COVID group [29, 30]. This was true even after controlling for surgery type, important to our population as RYGB is associated with greater weight loss [31–33]. Prior studies have suggested improved weight loss with increased follow-up rates, but this finding is not definitive [28, 34, 35]. Weight loss is multifactorial, making it difficult to pinpoint an exact cause for this difference. Thus, further studies are needed to identify these factors so that practice patterns promoting increased weight loss can be maintained.

Like our previously reported pre-COVID cohort, post-COVID LT and HT patients were similar in preoperative characteristics [15]. Unsurprisingly, post-COVID LT patients had 30% greater rates of public insurance compared to post-COVID HT and 15% greater rates of public insurance compared to pre-COVID LT groups. This is, again, attributable to the insurance expansion discussed above which is more likely to impact lower SES patients. Although post-COVID patients as a whole had longer wait time to surgery, there was no difference in wait time between LT and HT subgroups.

Importantly, prior to the pandemic, LT patients were half as likely to be discharged home on POD 1, 50% more likely to have any complication, and over twice as likely to be readmitted after discharge [15]. In the post-COVID era, these differences were no longer seen. This suggests that post-COVID protocols have evened the playing field for patients of lower SES, even with the added burden of the global pandemic. Furthermore, these findings reiterate the fact that despite the disparities in resources, LT patients benefit just as much from MBS as patients from more prosperous communities.

## Limitations

This study has several limitations, including its retrospective nature. What is more, implementation of telehealth follow-up visits necessitates self-reported weights. This is often measured on a home scale and cannot be independently verified. Nonetheless, studies have shown close fidelity of self-reported weights to clinic measured weights, making self-reported weight a viable proxy [36, 37]. This study is also limited in its single institution setting. While all protocols have a common framework, each hospital has its own policies and a single institutional study may mask the effect of differing practice patterns. Larger, multi-institutional studies should be conducted to confirm our results. Likewise, while patients are stratified to pre- and post-pandemic cohorts, the precautions and limitations imposed early in the pandemic have largely changed with time and abatement of the virus. Therefore, early post-COVID era may be different from late post-COVID era, with subsequent impact on patient care. Thus, as we transition away from the immediate post-pandemic era amidst continued healthcare evolution, future studies are needed to identify disparities and determine MBS protocols that optimize outcomes for patients of all SES.

## Conclusion

To our knowledge, this is the first study evaluating both post-pandemic outcomes and the changes in socioeconomic disparities in patients undergoing bariatric surgery. Health care changes in the wake of the pandemic have resulted in fewer complications and improved weight loss and follow-up. Most importantly, disparities seen among low tier patients prior to the pandemic were no longer seen in the post-COVID era. Thus, post-pandemic changes have had a protective effect on our patient population, especially among socioeconomically disadvantaged patients. This highlights the power of positive MBS protocol changes and the ability to create an even playing field for patients of all SES groups.
